# Thyroid Storm, a Mimicker of Guillain-Barre Syndrome: A Case Report

**DOI:** 10.7759/cureus.9918

**Published:** 2020-08-21

**Authors:** Aizaz Ali, Waseem T Malik, Umar Raja, Fahd A Khan, Irum Javaid

**Affiliations:** 1 Neurology, Shifa International Hospital, Islamabad, PAK; 2 Endocrinology, Shifa International Hospital, Islamabad, PAK; 3 Acute Medicine, James Cook University Hospital, Middlesbrough, GBR; 4 Anatomy, Northwest School of Medicine, Peshawar, PAK

**Keywords:** thyroid storm, thyrotoxicosis, guillain-barre syndrome, gbs, weakness, neuropathy

## Abstract

A 60-year-old woman with diabetes mellitus presented to the emergency department (ER) with complaints of lower limb weakness, preceded by diarrhea. She complained of sweating and palpitations. There was no fever, cough, trauma, seizures, or headache. There was global weakness in all four limbs with absent reflexes and hypotonia. Examination of cranial nerves, the sensory system, and other systems was normal. Guillain-Barre syndrome (GBS) was suspected, but due to the patient’s co-morbidities, treatment was withheld for 24 hours and the patient was kept under observation. Medical consultation was sought and thyroid function tests were ordered which showed thyroid-stimulating hormone (TSH) 0.019 uIU/ml (normal: 0.35-4.94 uIU/ml), free triiodothyronine (T3) 11.94 pg/ml (normal: 2.0-4.4 pg/ml), and free thyroxine (T4) >5 ng/dl (normal: 0.70-1.48 ng/dl). Thyroid storm was suspected and she was treated with hydrocortisone, propylthiouracil, Lugol iodine, and beta-blocker and her symptoms improved in 10 days with resolution of the weakness, confirming the diagnosis. Besides highlighting this association, this report demonstrates the importance of conducting thyroid function tests in patients presenting with axonal neuropathy. In patients having weakness in all four limbs and presenting with multiple comorbidities, we need to exclude medical reasons before starting treatment for GBS, such as in our case where it was thyrotoxicosis.

## Introduction

Thyroid storm is also known as thyrotoxic crisis. It is a life-threatening complication of hyperthyroidism and presents with sudden multi-system involvement [1]. It usually presents with symptoms of fever and altered mental status [2]. Cardiovascular involvement (tachycardia, arrhythmia, heart failure with pulmonary edema) and central nervous system involvement (anxiety, agitation, delirium, psychosis) is also usually present [3]. Some cases have been reported where thyroid storm has presented with weakness and paralysis. One such condition is called thyrotoxic periodic paralysis (TPP), which occurs due to low potassium levels and resolves after administration of potassium [4]. Our patient had normal potassium level of 4.50 mEq/L at baseline (normal: 3.5-5.0 mEq/L) and therefore did not have TPP.

This patient is a unique case of thyroid storm presenting as Guillain-Barre syndrome (GBS).

## Case presentation

A 60-year-old lady, known case of diabetes mellitus well controlled with glucophage, came to the ER with complaints of lower limb weakness for five days and vomiting and diarrhea for the past three days. The diarrhea was watery and did not contain any mucus or blood. The patient also reported one episode of per-rectal bleeding. She had no history of fever, cough, urinary incontinence, seizures, sensory loss, falls, or headache. She had also been previously hospitalised for similar complaints. Her Glasgow Coma Scale (GCS) was 15/15 and pupils were bilaterally equal and reactive. There was decreased tone in both lower limbs. Planters were down going bilaterally and reflexes were absent. Power was 3/5 in the right and left proximal upper limb and 4/5 in the right and left distal upper limb. Similarly, power was 2/5 in the right and left lower limbs, both proximally and distally. There was no sensory impairment and no facial deviation or deviation of the tongue. The rest of her physical examination was unremarkable. Baseline tests were ordered - haemoglobin (Hb) 10.6 g/dL (normal: 13.5-17.5 g/dL), potassium (K) 4.5 mEq/L (normal: 3.5-5 mEq/L), blood sugar random (BSR) 208 mg/dL (normal: <200 mg/dL), C-reactive protein (CRP) 12 mg/L (normal: 0-10 mg/L), and calcium (Ca) 7.54 mg/dL (normal: 8.4-10.2 mg/dL). Liver function tests (LFTs) and renal function tests (RFTs) were normal.

Her nerve conduction studies (NCS) were performed the next day and it showed findings suggestive of a moderate sensory motor axonal polyneuropathy affecting the upper limbs more than the lower limbs. The patient had also sweating and tachycardia, which were thought of as autonomic manifestations of Guillain-Barre syndrome (GBS). Although GBS was suspected, due to the patient’s comorbid conditions, it was decided to monitor the patient for 24 hours and to defer treatment for that duration. Thyroid function tests were ordered (24/February/2018) and the results came back abnormal, i.e., thyroid-stimulating hormone (TSH) 0.019 uIU/ml (normal: 0.35-4.94 uIU/ml), free triiodothyronine (T3) 11.94 pg/ml (normal: 2.0-4.4 pg/ml), and free thyroxine (T4) >5 ng/dL (normal: 0.70-1.48 ng/dL). Thyroid storm was confirmed and she was started on hydrocortisone, propylthiouracil (PTU), lugol iodine, and beta-blocker.

The patient was monitored and symptomatic improvement was seen in three to four days. Although the diarrhea persisted, the weakness in the limbs improved. Upon examination, power in the right and left upper limbs was 4/5, and power in the right and left lower limbs was 2/5 and 4/5, respectively. Leucocytes were 14,500/mm^3^ (normal: 4,500-11,000/mm^3^), Hb 10.8 g/dL (normal: 13.5-17.5 g/dL), and platelets 126,000/mm^3 ^(normal: 150,000-400,000/mm^3^). Electrolytes were in the normal range.

In a week’s time, the patient was significantly well. On examination, her GCS was 15/15. Speech and comprehension was normal, and she was moving all four limbs. She was referred to the endocrinology department and prepared for discharge. Intravenous hydrocortisone was changed to tablet prednisolone 20 mg twice daily, and PTU, Lugol iodine, and propranolol (beta-blocker) were continued. The patient was told to follow up with thyroid function test (TFT) results after a week.

The patient came for follow-up with her TFT results on 12/March/2018 - TSH 0.01 uIU/ml (normal: 0.35-4.94 uIU/ml), free T4 1.43 ng/dL (normal: 0.70-1.48 ng/dl), and T3 70.9 ng/dl (normal: 58-159 ng/dl). Lugol iodine and propranolol were discontinued, dose of PTU was reduced to 200 mg twice daily, and the patient was referred for radio-iodine treatment. Repeat testing a month later on 10/April/2018 showed TSH 0.01 uIU/ml (normal: 0.35-4.94 uIU/ml), free T4 0.90 ng/dL (normal: 0.70-1.48 ng/dL), and T3 232.8 ng/dl (normal: 58-159 ng/dl). The dose of PTU was increased and gliclazide was discontinued. The patient was subsequently followed up in the endocrinology department in the coming months with serial TFTs and adjustment of her anti-thyroid medications were made until her levels normalised.

The following table illustrates the levels of TSH, FT3, and FT4 during the time period the patient was being followed up and dose adjustments were being made (Table 1).

**Table 1:** Serial measurement of thyroid function tests during treatment TSH: Thyroid-stimulating hormone; FT3: Free triiodothyronine; FT4: Free thyroxine.

	24-Feb-18	05-Mar-18	12-Mar-18	10-Apr-18	Normal Reference Range
TSH	0.01 uIU/ml	0.01 uIU/ml	0.01 uIU/ml	0.01 uIU/ml	0.35-4.94 uIU/ml
FT3	11.94 pg/ml	140 ng/dl	70.9 ng/dl	232.8 ng/dl	58-159 ng/dl
FT4	>5 ng/dl	2.88 ng/dl	1.43 ng/dl	0.90 ng/dl	0.70-1.48 ng/dl

## Discussion

Thyroid storm, also known as thyrotoxic crisis, is a rare life-threatening exacerbation of hyperthyroidism, characterized by evidence of decompensation in one or more organ systems. Its mortality ranges from 8%-25% and therefore prompt recognition and treatment is essential [3,5]. It is usually triggered by precipitants such as infection, trauma, surgery and myocardial infarction. In some cases, exposure to iodinated contrast and non-compliance with anti-thyroid medication has also led to thyroid storm [6].

Thyroid storm presents with a myriad of symptoms, including but not limited to fever, altered mental status, cardiovascular symptoms (tachycardia, arrhythmia, heart failure with pulmonary edema) and central nervous system symptoms (anxiety, agitation, delirium, psychosis) [2, 3].

It is diagnosed by a combination of biochemical laboratory tests confirming thyrotoxicosis, along with the life-threatening symptoms of hyperthyroidism. Several scoring systems have been introduced to diagnose thyroid storm such as the Burch-Wartofsky scoring (BWS) system and the Akamizu criteria [6]. The BWS scoring system consists of temperature, tachycardia, presence of atrial fibrillation, heart failure, gastrointestinal (GI) dysfunction, and the presence of precipitating factor. A total score of more than 45 is highly suggestive of thyroid storm, 25 to 44 supports the diagnosis, and less than 25 makes the diagnosis unlikely [3]. Our patient had a score of 55, and therefore a diagnosis of thyroid storm was confirmed.

Treatment consists of multiple methods to control various aspects of the thyroid storm. Supportive measures such as oxygen, intravenous fluids and cooling blankets are employed, and any underlying precipitating causes are addressed. Anti-thyroid drugs such as methimazole or propylthiouracil are used to decrease the production of thyroid hormones. Beta-blockers e.g. propranolol are used to prevent adrenergic manifestations such as tachycardia. A potassium iodide solution is employed to decrease thyroid hormone release from the thyroid, and steroids are used to decrease the peripheral conversion of T4 to T3 [3,6,7].

This case report describes a patient that presented with decreased strength in the lower limbs preceded by episodes of diarrhea and vomiting. There was a loss of tone and reflexes in the lower limbs, and an absence of any sensory or cranial nerve abnormalities. Nerve conduction studies demonstrated moderate sensory motor axonal polyneuropathy affecting the upper limbs more than the lower limbs. These findings were consistent with GBS. The patient also reported sweating and tachycardia, which could both be an autonomic manifestation of GBS or a symptom of thyroid storm.

Some other causes of acute polyneuropathy apart from hyperthyroidism consist of thyrotoxic periodic paralysis (TPP), polio, hepatitis B, tick paralysis, botulism, and heavy metal poisoning. TPP was excluded due to the lack of hypokalaemia at baseline, absence of muscle cramps in the lower limbs, and nerve conduction studies (NCS) not typical of TPP [8]. The patient had negative serology for hepatitis B, and had received the polio vaccine in childhood so they were also excluded as causative factors. Tick paralysis was excluded because it has not been reported in Pakistan to the authors' best knowledge, and botulism was excluded because it presents with descending paralysis and cranial nerve palsies, symptoms which were not present in our patient. The patient’s history excluded heavy metal poisoning.

Polyneuropathy has been previously described in literature in association with thyrotoxicosis. In fact, paraplegia-like weakness during severe hyperthyroidism has been described by Charcot as early as 1888 [9]. The exact underlying mechanism for the association between the two phenomenon is still not well understood. Pandit et al. revisited the Basedow’s paraplegia and stated that the neurological dysfunction can be either due to an excess surge of thyroid hormone in the circulation or due to an immune-mediated process [10]. Duyff et al. concluded at the end of their studies that muscle weakness in hyperthyroidism may be due to a functional muscle weakness rather than a myopathy. They also demonstrated a statistically significant correlation between the level of mean FT4 concentrations and the presence of paresis [11]. Another study conducted by McComas et al. on 20 patients also provided good reasons for identifying a neuropathic lesion as being responsible for the “so-called” myopathic features of thyrotoxicosis [12].

The pathogenesis of neuropathy in hyperthyroid states is still not well understood and further studies are required to investigate it. A high index of suspicion for thyroid dysfunction is necessary when evaluating a patient with polyneuropathy and routine thyroid function tests should be ordered when evaluating a patient with acute polyneuropathy.

## Conclusions

GBS is an inflammatory disease usually after diarrhea or respiratory illness. It is associated with lower motor neuron disease and sometimes with autonomic dysfunction. It is treated with plasma exchange. Thyrotoxicosis is a medical emergency. It is associated with apprehension, sweating, tachycardia, weakness, and cardiovascular and central nervous system symptoms. It is treated with a combination of anti-thyroid drugs, steroids, beta-blockers, and Lugol iodine. GBS and thyrotoxicosis can both present with weakness and therefore we need to judge patients clinically as nerve conduction studies can be abnormal in both.
